# Michigan State Dental Association

**Published:** 1885-06

**Authors:** E. C. Moore


### MICHIGAN STATE DENTAL ASSOCIATION.

Reported for the Independent Practitioner, by E. C. Moore, D. D. S.

(Concluded from page 269.)

*Dr. Shattuck*, of Detroit—It seems to me that if amalgam is such an atrocious filling, disastrous results would have been noticed before this time. It has been used for a good many years. I have practiced for twenty-five years and used it, and I have not observed any serious injury from it. I want statistics, and then if it can be shown that patients are being injured by it I will stop using it, but I require certain proof before I do that. I can see that amalgam may do some harm in young teeth of a loose structure; it might be carried into and destroy the pulp; but in teeth as we usually find them, and using amalgam in a careful manner, there is no more danger of destroying the pulp by using an amalgam filling than with any other metal. There is too great a tendency in the profession, or with some members of it, to attribute every dead tooth that has an amalgam filling to the amalgam itself. I want statistics.

*Dr. Douglass*—I have used Cocaine in operating about exposed pulps, and have found that the patients suffer little pain, sometimes none at all. I have used it on the gums before the application of the rubber dam, and have found that the patient felt no sensation of pain. I have also tried Cocaine in a number of cases, to prevent pain from cutting sensitive dentine, but I cannot say that I have had any success with it in such cases.

*Dr. Long*, of Monroe—I cannot speak favorably of Cocaine. I have tried it, and have not been able to extract a pulp yet with decreased sensation. As far as extracting teeth is concerned, I have tried it in a number of cases and failed to get good results. I have injected it around the gum, and up under the gum, and I have waited five minutes, and fifteen minutes, without results.

*Dr. Cowie*, of Detroit—I have had some experience with Cocaine. Two or three days ago I applied a four per cent solution to an inflamed gum, very susceptible to the touch, where I wished to drill through the process to give vent. I allowed the Cocaine to remain for five minutes, and then went through the gum and process with my drill, and there was no pain. I have also had some experience in removing pulps, where I applied the same solution of Cocaine, and there appeared to be very little, if any, pain, but I have had no success whatever with Cocaine in the extraction of teeth; the pain of extraction was not reduced at all.

*Dr. Moore*, of Detroit—I have not had any good results from Cocaine. I have used it in various solutions, with the teeth dry, and with them wet, with and without the rubber dam, and in teeth with the pulps in all degrees of inflammation, exposure and disease, and the only result that I have noticed is that they seem to be more sensitive than before. I have used it for from five minutes to an hour and a half, with the same result. I have applied it to sensitive dentine, and to exposed pulps, and to teeth where a part of the pulp had been removed while a portion was yet alive in the root further down. I have tried it in every way, and so far without any satisfactory effects. It may be that the Glover preparation is better than the others. I will give it a trial.

*Dr. Clawson*, of Detroit—I have tried Cocaine without any good effects, but I have been able to get good results from a strong alkali mixed with carbolic acid. I am satisfied that the preparation of Dr. Robinson, of equal parts caustic potash and carbolic acid, will prove one of the best things for sensitive dentine that we have had. I have hitherto used a somewhat different preparation; mine is not the caustic potash, but simply the carbonate, but I can readily see that the caustic would be better than the carbonate, when mixed with carbolic acid.

*Dr. Harroun*—We are a body of experimenters, as a society, and have been at it for a great many years. The rapid strides that the profession has made for fifty years, show a great deal of progress, and we will, by and by, accomplish much in this direction, in addition to what we have already done. Naturally, we make mistakes. I thought Cocaine was just what the profession needed, but it does not benumb the pulp, and so far is a failure; but the gums, immediately around the teeth in which it is used, will be so benumbed that we may be able to use an instrument sufficiently sharp to burnish gutta-percha, without causing any pain whatever, but the moment the pulp is touched intense pain is caused. As to the Robinson remedy that has been mentioned, it is not as good when first prepared as after it has stood a few days. It can be applied to an exposed pulp without injuring it a particle. I do not fill a tooth without putting that in. It causes a sharp pain at first, but that soon passes away, and the patients are willing to submit to a short pain if the excavation and operation can be carried on painlessly afterwards. It coagulates the albumen of the tubuli, and the tooth is in better condition for filling. In applying these things a great deal depends upon the patient. The tissues of one will absorb a remedy, while those of another will resist it. These differences in patients must be taken into account. I have used the Robinson remedy for Pyorrhœa, with success, but Pyorrhœa cannot be cured unless the teeth are first thorougly cleaned. The granulations of tartar must be removed, and then when the Robinson remedy is applied it acts as a cauterant; it contracts the pocket around the tooth, and from one to four applications will effect a cure. I apply it on floss cotton, poking a little of it down with an instrument. I have had fair success with Dr. Harlan's method, but nothing compared with that which I have had with the Robinson remedy.

